# O021. Abnormal connectivity within executive resting-state network in migraine with aura

**DOI:** 10.1186/1129-2377-16-S1-A156

**Published:** 2015-09-28

**Authors:** Antonio Russo, Francesca Conte, Laura Marcuccio, Fabrizio Esposito, Alfonso Giordano, Manuela De Stefano, Mario Cirillo, Alessandro Tessitore, Gioacchino Tedeschi

**Affiliations:** Department of Medical, Surgical, Neurological, Metabolic and Aging Sciences, Second University of Naples, Naples, Italy; MRI Research Center SUN-FISM, Second University of Naples, Naples, Italy; Institute for Diagnosis and Care “Hermitage Capodimonte”, Naples, Italy; Department of Medicine and Surgery, University of Salerno, Baronissi, (SA) Italy; Neuroradiology Unit, Department of Clinical and Experimental Medicine and Surgery, Second University of Naples, Naples, Italy

## Background

Despite the fact that the clinical features of migraine are well described, the relationship between migraine and cognitive performance is still poorly understood. Indeed, some authors have reported the presence of cognitive deficits in patients with migraine without aura (MwoA) and with aura (MwA) whereas others have not confirmed these findings. Although neuropsychological studies in migraine are not conclusive, the most likely pattern of neuropsychological impairment would relate to the cognitive domain of executive functions (EF) [1]. Recent imaging studies have shown a significant functional connectivity decrease within the fronto-parietal networks (FPN), known to be associated with EF, in patients with MwoA in absence of significant executive dysfunction [2].

## Objective

To further explore FPN functional connectivity in patients with MwA and patients with MwoA, in the interictal period.

## Methods

Using resting-state functional magnetic resonance imaging (RS-fMRI), we compared functional connectivity within the FPN in 20 patients with MwA, versus 20 sex- and age-matched healthy controls (HC). To examine the specificity of any observed differences in FPN functional connectivity between patients and HC, we further studied 20 age- and sex-matched patients with MwoA. Furthermore, we assessed the correlation between functional connectivity within FPN and EF in both migraine groups. Finally, we used voxel-based morphometry to assess whether between-group differences in functional connectivity were dependent on structural differences.

## Results

Neuropsychological data revealed no significant executive dysfunction in both migraine groups compared to HC. RS-fMRI showed that both MwA and MwoA patients, compared to HC, had a significant functional connectivity decrease within the right FPN and specifically in the middle frontal gyrus and the dorsal anterior cingulate cortex. There were no structural differences between the three groups.

## Conclusions

Our data demonstrate that, even in the absence of clinically evident EF deficits, MwA and MwoA are associated with reduced FPN functional connectivity. We suggest that disrupted FPN functional connectivity might be only a part of a complex cascade that terminates in a migraine attack. In this context, FPN abnormalities may be the neuronal substrate on which biological, genetic and environmental factors could induce, and in turn correlate with, migraine attacks mostly characterized by high pain intensity in patients with MwoA and aura phenomenon in patients with MwA. In other terms, observed FPN connectivity changes may represent a migraine biomarker, probably related to well-known maladaptive stress response in migraine patients.

Written informed consent to publish was obtained from the patient(s).
